# Case of Hydrothorax

**Published:** 1814-08

**Authors:** W. Wardley

**Affiliations:** Warren-Street


*For the Medical and Physical Journal.*

### *Case of Hydrothorax;* by Mr. Wardley.

JOHN WHITE had a dry cough, with extreme difficulty of breathing, which prevented his lying down in bed. He was troubled with distressing palpitations of the heart, and complained of a coldness and weight in the stomach. The lips were pale; he was drowsy, though never refreshed by sleep; his appetite was lost; he was generally thirsty; and the pulse was strong, without perceptible intermission. The only remedy employed was quicksilver pill with squills, a blister to the chest, and nitre in his common drink. In ten days he was evidently better; and in six weeks entirely recovered.

W. WARDLEY.

*Warren-Street.*

